# Thoraco-Omphalopagus Conjoined Twins: A Rare Presentation in the Dominican Republic

**DOI:** 10.7759/cureus.58369

**Published:** 2024-04-16

**Authors:** Paria Remolina, Yoalkris E Salcedo, Walid A Malik, Katy Rodriguez, Rosanda Sanchez

**Affiliations:** 1 Surgery, Universidad Nacional Pedro Henríquez Ureña, Santo Domingo, DOM; 2 Surgery, Universidad Iberoamericana, Santo Domingo, DOM; 3 Medicine, Universidad Nacional Pedro Henríquez Ureña, Santo Domingo, DOM; 4 Neonatology, Hospital Docente Nuestra Señora de la Altagracia, Santo Domingo, DOM

**Keywords:** pediatric intensive care unit, prognosis, fetal echocardiogram, neonatal examination, heterotaxy, syphilis, dominican republic, cesarean section, thoraco-omphalopagus, conjoined twins

## Abstract

The case involves a 23-year-old Dominican woman's admission to Hospital Docente Nuestra Señora De la Altagracia for an elective cesarean section at 38 weeks gestation with conjoined twins. Despite effective treatment for syphilis in the third trimester, her medical history complicated the situation. The twins, thoraco-omphalopagus conjoined, share vital organs and exhibit congenital anomalies, posing unique diagnostic and management challenges. This case contributes to the scarce literature on conjoined twins, especially in the Dominican Republic. It highlights the complexities of diagnosis, prognosis, and management strategies for such rare cases. This emphasizes the importance of ongoing research and medical intervention in addressing these challenges.

## Introduction

The occurrence of conjoined twins presents a unique and medically intricate scenario in obstetrics, challenging both clinicians and researchers alike. On March 13, 2024, at 7:32 am, the Hospital Docente Maternidad Nuestra Señora De la Altagracia encountered such a case as a 23-year-old primigravida Dominican woman, who had received prenatal care, was admitted for an elective cesarean section at 38 weeks gestation, carrying conjoined twins. This case adds to the limited body of literature surrounding this rare phenomenon. The mother's medical background revealed a blood group of A Rh negative, with an absence of antenatal, congenital, or neonatal fatalities. Additionally, she had a positive diagnosis of syphilis in the third trimester and was diagnosed with severe preeclampsia during her antenatal care.

Upon examination, the twins were confirmed to be thoraco-omphalopagus conjoined twins, sharing vital organs and presenting with congenital anomalies. This condition occurs in approximately 1 in every 50,000 pregnancies and presents unique challenges in diagnosis and management. Furthermore, the prevalence of conjoined twins, particularly thoraco-omphalopagus presentation, has garnered attention, with rare instances documented in the Dominican Republic. This case adds to the growing understanding of such occurrences, with previous cases documented at the same hospital. Understanding the etiology and prognosis of conjoined twins is crucial for appropriate management and counseling of affected families. This report delves into the intricacies of the case, shedding light on the challenges faced by both medical professionals and families in such rare and complex situations.

## Case presentation

Maternal history and delivery

**Figure 1:**
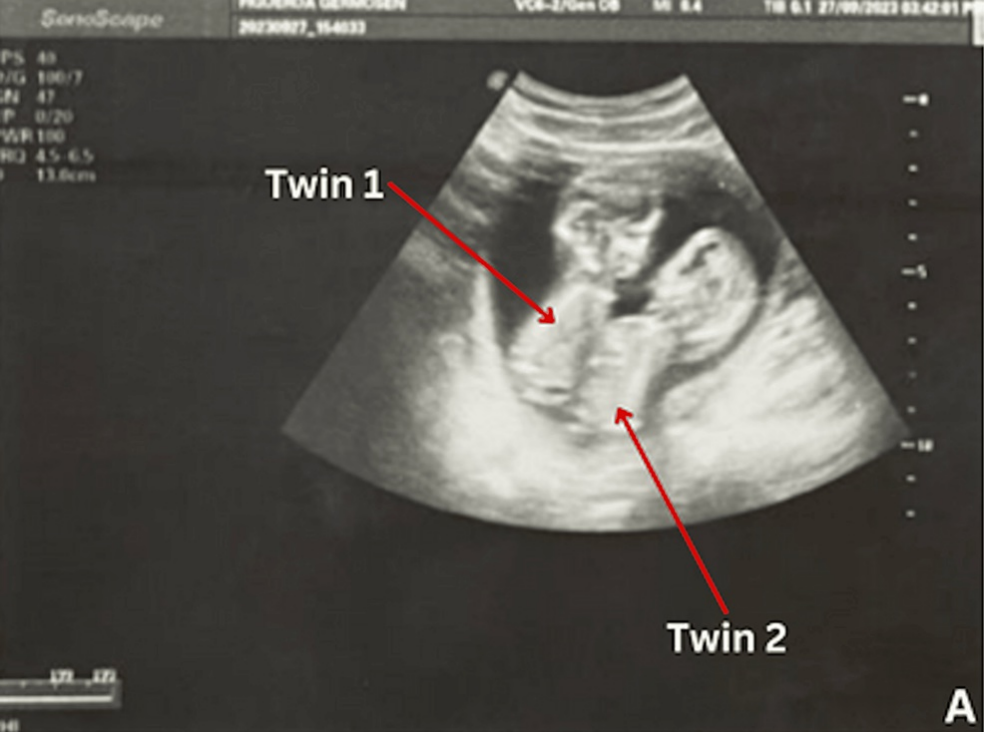
Prenatal sonography image showing the union between both fetuses at thoracic level and upper abdomen, sharing a single heart, showing total separation of heads (located face to face), pelvis and upper and lower limbs. The patient was diagnosed at this point with a twin gestation of thoraco-omphalopagus conjoined twins at 14.2 weeks.

On March 13, 2024, at 7:32 am, a 23-year-old primigravida of Dominican descent was admitted to the esteemed Hospital Docente Maternidad Nuestra Señora De la Altagracia, presenting at 38 weeks gestation with a rare and complex case of conjoined twins, necessitating an elective cesarean section. Notably, prenatal ultrasonography at 14 weeks gestation had already indicated the presence of conjoined twins, as seen in Figure 1, adding an early layer of complexity to the pregnancy journey. The patient's medical history revealed a blood group of A Rh negative, while specific details regarding her last menstrual period (LMP) were not documented. Importantly, there were no antecedents of antenatal, congenital, or neonatal deaths, and the absence of significant medical conditions such as rubella, measles, and chickenpox, among others, was confirmed. However, a notable aspect of her medical history included a positive diagnosis of syphilis in the third trimester, for which she received comprehensive treatment consisting of three doses of benzathine penicillin. Additionally, the patient had been diagnosed with severe preeclampsia during her antenatal care, further complicating the intricacies of her management.

Furthermore, the patient adamantly denied any engagement in toxic habits such as alcohol, tobacco, or illicit drug use. Upon examination, she presented as conscious and stable, despite the diagnosis of severe preeclampsia, with vital signs within normal parameters, including a blood pressure of 120/80 mmHg and a fetal heart rate (FHR) of 142 beats per minute. Noteworthy was the absence of edema. Vaginal examination revealed no cervical dilation, confirming a cephalic presentation and the absence of signs of labor or rupture of membranes.

The subsequent cesarean section proceeded uneventfully, revealing upon examination a sizable placenta devoid of calcifications, alongside an elongated umbilical cord devoid of any discernible abnormalities such as circular wrapping or vascular irregularities.

Pre-birth cardiac findings

Preceding birth, ultrasound examinations unveiled compelling insights into the cardiac status of the conjoined twins. Noteworthy was the cardio/thoracic ratio, which measured at 62%, exceeding the established normal range of 35 to 55%, indicative of cardiomegaly. Twin 1 exhibited a diagnosis of heterotaxy, a condition characterized by the atypical mirror-image arrangement of organs within the chest and abdomen. Particularly striking was the identification of a solitary atrium with left-sided appendages, diverging from the typical bilateral atrial configuration. Furthermore, the ultrasound revealed the presence of only one functional left ventricle, accompanied by residual elements of the right ventricle. Significant valvular insufficiency was observed in the atrioventricular valve apparatus, alongside a notable ventricular septal defect spanning the left and right atria.

In addition to these findings, the ultrasound delineated a heart rate ranging between 132 to 138 bpm, consistently falling within the established normal range. Anatomically, the cardiac examination unveiled a univentricular heart with left morphology, featuring a pulmonary artery branching into two distinct branches, with the left branch seamlessly merging with the ductal arch. Notably, bifurcation of the descending aorta was also documented, underscoring the intricacies of the twins' cardiac anatomy.

Neonatal examination

**Figure 2:**
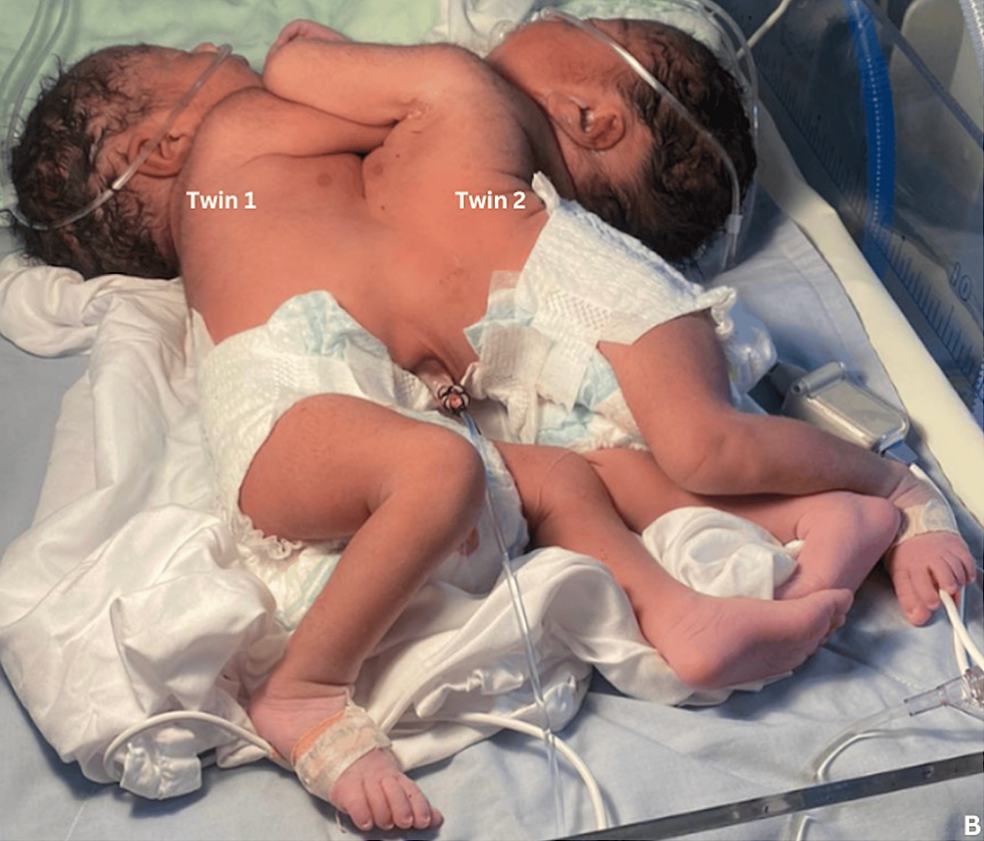
Photographic image shows the thoraco-omphalopagus conjoined twins one day after delivery.

**Figure 3:**
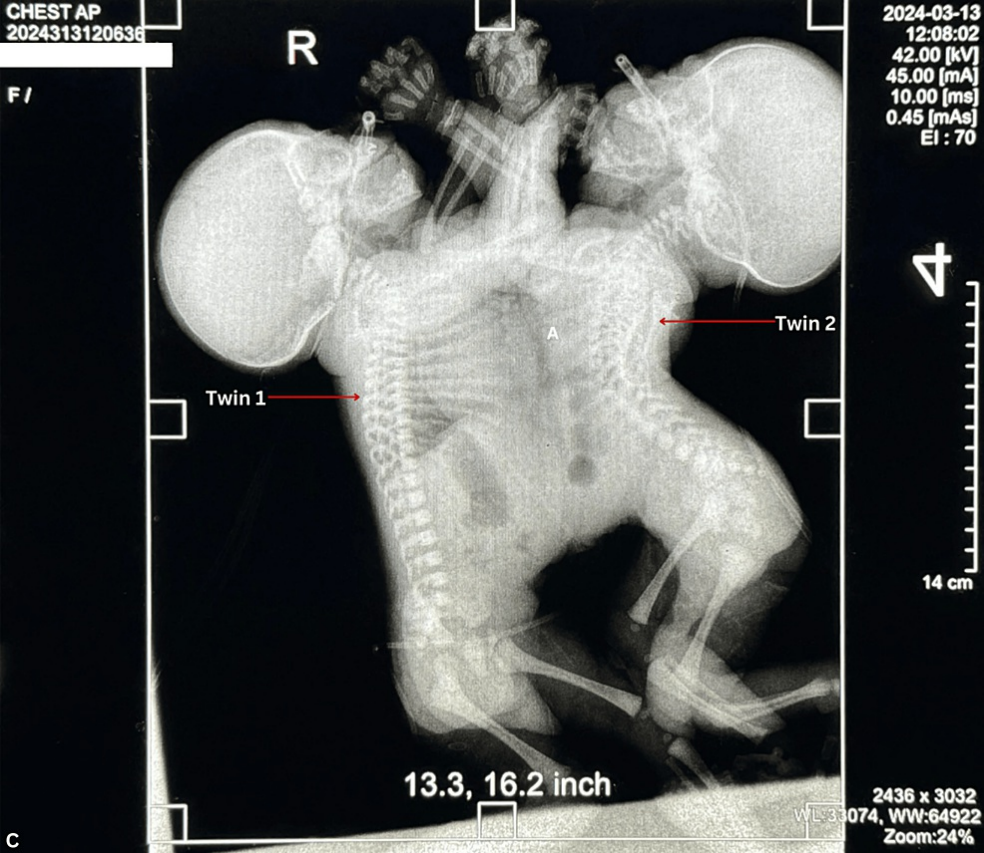
X-ray imaging of the Anteroposterior (AP) view of the thoracic cavity, demonstrating the common hepatic and cardiac structures shared by both twins, alongside their respective individual gastrointestinal tracts.

Upon birth, the comprehensive neonatal assessment revealed the conjoined twins to be at a gestational age of 38 weeks. Both infants were identified as female, with a weight of 10 pounds and 16 ounces (4,536 grams). Twin 1 exhibited a length of 44.0 cm, while twin 2 measured 39.0 cm. Cranial perimeter (CP) measurements were recorded at 33.0 cm for twin 1 and 32.0 cm for twin 2, with a combined chest perimeter (PT) of 42.0 cm. Notably, APGAR scores were documented at 7 at 1 minute and 8 at 5 minutes for twin 1, whereas twin 2 scored 6 at 1 minute and 7 at 5 minutes. Upon auscultation, a pan-systolic and diastolic murmur was discerned during the cardiac examination. The lungs of each twin were ventilated with findings of pulmonary respiratory difficulty in both conjoined twins requiring oxygen via nasal CPAP, maintaining an oxygen saturation of 70-80%. The abdomen displayed conjoined features with a single umbilical cord was visualized and an MRI was performed, which is pending. Anal patency was confirmed, and the genitalia were deemed appropriate for their sex and age. Plantar folds exhibited 2/3 anterior creases for both twins, with no evidence of desquamation. Clinically, the twins were diagnosed as thoraco-omphalopagus conjoined twins, characterized by their fusion at the level of the thorax and abdomen as seen in Figures 2, 3.

Following delivery, the twins were promptly admitted to the neonatal intensive care unit (NICU), where they received specialized care. A comprehensive fetal echocardiogram confirmed the presence of complex congenital heart disease in both infants. Prophylactic administration of Ampicillin and Amikacin was initiated based on abnormal hematological findings, including an abnormally elevated white blood cell count (Table 1).

**Table 1:** Post-birth neonatal lab findings WBC = White Blood Cell Count; RBC = Red Blood Cell Count; HGB = Hemoglobin; HCT = Hematocrit; MCV = Mean Corpuscular Volume; MCH = Mean Corpuscular Hemoglobin; MCHC = Mean Corpuscular Hemoglobin Concentration; PLT = Platelets; RDW-SD = Red Cell Distribution Width-Standard Deviation; RDW-CV = Red Cell Distribution Width-Coefficient of Variation; PDW = Platelet Distribution Width; MPV = Mean Platelet Volume.

Laboratory	Result	Reference Range
Hemogram		
WBC	20.62	3.50 - 9.50 x 10^3^/uL
RBC	4.70	3.80 - 5.80 x 10^6^/uL
HGB	18.6	11.5 - 17.5 g/dL
HCT	53.2	35.0 - 50.0%
MCV	113.1	82.0 - 100.0 fL
MCH	39.7	31.6 - 35.4 pg
MCHC	35.1	31.6 - 35.4 g/dL
PLT	245	125 - 350 x 10^3^/uL
RDW-SD	65.6	35.0 - 56.0 fL
RDW-CV	16.9	11.5 - 45.0%
PDW	7.0	9.0 - 17.0 fL
MPV	8.0	7.0 - 11.0 fL
Differential		
Neutrophil %	36.0	50.0 - 70.0%
Mixed %	13.2	3.0 - 9.0%
Lymphocyte %	50.8	3.50 - 9.50%
Neutrophil #	7.43	2.00 - 7.00 x 10^3^/uL
Mixed #	2.72	0.10 - 0.90 x 10^3^/uL
Lymphocyte #	10.47	1.10 - 3.20 x 10^3^/uL
Serology		
Direct Coombs Test	Negative	
Treponema pallidum PCR	Negative	
VDRL	Non-reactive	

## Discussion

Conjoined twins manifest in approximately 1 in 50,000 pregnancies and an infrequent occurrence of 1 in 250,000 live births as referenced from the Nelson Textbook of Pediatrics [1]. These exceptional beings emerge as obligate monozygotes, stemming theoretically from the subsequent fission of a singular zygote or the fusion of two distinct zygotes [2]. Intriguingly, this phenomenon tends to manifest more prevalently within the female demographic, a peculiarity deserving of a detailed investigation into its underlying percentage (Osmanağaoğlu et al., 2011) [3].

The prognosis of conjoined twins is uncertain, as it depends on the feasibility of surgical separation, which is influenced by the extent of vital organ sharing between them. The sites of connection among these unique individuals vary, encompassing thoraco-omphalopagus, thoracopagus, omphalopagus, craniopagus, and incomplete duplication, with thoraco-omphalopagus reigning as the predominant presentation (Mutchinick et al., 2011) [4], as exemplified in our particular case.

The genesis of conjoined twins traces back to the incomplete division of a solitary zygote roughly 12 to 15 days post-fertilization, thereby leading to the intertwining of fetal structures. This process aligns with the classical fission theory, wherein twinning unfolds through the division of a singular conceptus, resulting in divergent amnionicity and chorionicity. However, the fission theory's explanatory power falters in the face of various atypical twinning occurrences, including diamniotic dichorionic monozygotic twinning post-single-embryo transfer during the late blastocyst stage, phenotypically discordant monozygotic twins, and asymmetrically attached conjoined twins. To address these anomalies, an alternative fusion theory of twinning has been posited, wherein the inner cell masses of trophectoderm fuse after the initial 2-cell splitting stage. Additionally, the fusion theory proposes another avenue wherein initially discrete monovular embryonic discs unite secondarily, yielding conjoined twins.

The advent of monozygotic twins appears to elude direct heritable factors [4]. However, a correlation has been observed with the employment of reproductive technologies, with heightened occurrences of both monozygotic and dizygotic twinning.

## Conclusions

The case underscores the intricate and formidable nature of thoraco-omphalopagus conjoined twins, marked by an uncertain and potentially dire prognosis. There is a conjecture that both twins may face mortality owing to their shared single heart. Presently, they are under meticulous observation in the neonatal intensive care unit at Hospital Docente Maternidad Nuestra Señora De la Altagracia, receiving comprehensive medical attention tailored to their complex needs. Notably, thoraco-omphalopagus twins have been increasingly documented at Hospital Docente Maternidad Nuestra Señora De la Altagracia in the Dominican Republic. Specifically, two cases have been recorded: one involving a pair of boys born in July 2023, and the other featuring boys born in August 2023. The current case involves girls born in March 2024.
